# Latent class analysis of actigraphy within the depression early warning (DEW) longitudinal clinical youth cohort

**DOI:** 10.1186/s13034-024-00843-8

**Published:** 2024-11-19

**Authors:** Lydia Sequeira, Pantea Fadaiefard, Jovana Seat, Madison Aitken, John Strauss, Wei Wang, Peter Szatmari, Marco Battaglia

**Affiliations:** 1 Centre for Child and Youth Depression Centre for Addiction & Mental Health, Toronto, ON Canada; 2 Faculty of Health - Department of Psychology, York University, Toronto, Canada; 3 Island Health, Victoria, BC Canada; 4 Department of Psychiatry, University of Toronto, Toronto, ON Canada; 5 Child Youth and Emerging Adult Programme, Centre for Addiction & Mental Health, 80 Workman Way, Toronto, ON M6J 1H4 Canada

**Keywords:** Depression, Adolescence, Actigraphy, Activity, Sleep, Latent Class Analysis

## Abstract

**Background:**

Wearable-generated data yield objective information on physical activity and sleep variables, which, are in turn, related to the phenomenology of depression. There is a dearth of wearable-generated data regarding physical activity and sleep variables among youth with clinical depression.

**Methods:**

Longitudinal (up to 24 months) quarterly collections of wearable-generated variables among adolescents diagnosed with current/past major depression. Latent class analysis was employed to classify participants on the basis of wearable-generated: Activity, Sleep Duration, and Sleep efficiency. The Patient Health Questionnaire adapted for adolescents (PHQ-9-A), and the Ruminative Response Scale (RRS) at study intake were employed to predict class membership.

**Results:**

Seventy-two adolescents (72.5% girls) were recruited over 31 months. Activity, Sleep Duration, and Sleep efficiency were reciprocally correlated, and wearable-generated data were reducible into a finite number (3 to 4) of classes of individuals. A PHQ-A score in the clinical range (14 and above) at study intake predicted a class of low physical activity (Acceleration) and a class of shorter Sleep Duration.

**Limitations:**

Limited power related to the sample size and the interim nature of this study.

**Conclusions:**

This study of wearable-generated variables among adolescents diagnosed with clinical depression shows that a large amount of longitudinal data is amenable to reduction into a finite number of classes of individuals. Interfacing wearable-generated data with clinical measures can yield insights on the relationships between objective psychobiological measures and symptoms of adolescent depression, and may improve clinical management of depression.

**Supplementary Information:**

The online version contains supplementary material available at 10.1186/s13034-024-00843-8.

## Introduction

Major depressive disorder (MDD) affects a significant proportion of citizens worldwide; studies carried out amongst children and adolescents convey estimates of both apparent recent increase [1], and relatively low prevalence of MDD [2], which in turn led some to reconsider the construct validity of the diagnosis of depression [3] and the allocation of risk factors (e.g., individual vs. societal [4], or socio-demographic vs. biological [5]) for the younger stratum of the population. In as much as younger individuals face novel environments and new hazardous factors compared to the previous generations, a deeper understanding of their living contexts and psychobiological landscapes becomes necessary. This can help to better frame the value and the meaning of the MDD diagnosis, for both classificatory and therapeutic reasons.

In addition to the current methods of diagnosing MDD through clinical assessments of mood, activity, sleep, and anhedonia, a host of new technologies can help map the construct more objectively, and with unprecedented density of information. Specifically, with the proliferation of smartphones, smartwatches and wearables, one can capture objective sensor data on sleep and activity, as well as time-locked information on emotions and cognitions among adult [6] as well as younger [7, 8] individuals. This large amount of raw data can then be converted into meaningful low-level behavioural markers (e.g. location type, activity type, sleep duration, phone usage), high-level behavioural markers (e.g. hedonic activity, fatigue, distractibility), and eventually affective states and clinical conditions (e.g. depression, anxiety), which in turn can be used to investigate aetiology, prognosis or treatment [9].

Specifically, physical activity and sleep can be mapped by portable actigraphs that are sensitive to acceleration/deceleration (hence the term ‘accelerometry’) in a three-dimensional space. For this reason, accelerometry is increasingly adopted to objectively quantify physical activity, sleep, and circadian rhythms in people with mood disorders.

More than 50 accelerometry-based controlled studies of MDD have now become available [10–15]: they consistently show lower and more variable daytime physical activity, poorer sleep efficiency, and dampened circadian rhythms associated with MDD. Moreover, analyses that covered two or more weeks of accelerometry data and compared acute vs. remitted MDD episodes, indicate the presence of trait- vs. state issues. Specifically, since alterations in physical activity and circadian rhythmicity appear to persist after remission and show familial aggregation, they may constitute trait markers of MDD; on the contrary, sleep difficulties undergo resolution after acute episodes, and may more likely reflect a state marker of MDD [12].

The host of new and dense data points that can be acquired by accelerometry bears at least three important implications. First, the adoption of technologies can improve the precision and reliability of data collection, and help address some challenges associated with MDD assessment, such as the subjective nature of diagnostic processes and the variability of information across informants (e.g., relatives, caregivers or parents vs. clients). The high correlation between mental health and changes in behaviour as detectable by digital devices supports the viability of these technology-assisted approaches [7]. Second, future, iterative processes of revision of diagnostic criteria may use the information obtained by sensor data and provide support to the analyses of which phenomena should be included -or left out- as core diagnostic elements, or peripheral associated features of mental disorders. Third -and possibly most importantly- several variables that are captured by sensors -such as physical activity- are modifiable factors that have potential, significant, bi-directional influences on MMD [16–18], so a dual role of clinical readouts and therapeutic tools can be envisaged [8, 17, 19, 20].

Turning to the specific context of adolescence, adolescent MDD (AMDD) presents with a tendency towards long, debilitating episodes, which makes the appraisal of time variability and prediction of relapse in the younger age range particularly relevant [1]. Because of the extensive dissemination of digital devices amongst youth and adolescents, and their familiarity with contemporary technologies, measuring and improving their mental health by digital phenotyping appears a viable approach, and a tool that can be integrated within the context of more traditional clinical approaches [7]. While a growing body of literature investigating affective states and/or physical activity through wearables in children and adolescents is becoming available, objective sensor-based data available for depression in childhood and adolescence remain scarce and based on non-clinical samples, with depression ascertained as a continuous variable, rather than a clinical category. A longitudinal study based on the large Avon Longitudinal Study of Parents and Children (ALSPAC)- general population cohort [21] showed the presence of 3 discrete trajectories of physical activity, and the association between sedentary activity and greater risk for depressive symptoms at 18 years of age. However, there is still a dearth of knowledge on wearable-derived data in clinical samples of children/adolescents diagnosed with AMDD, so that the temporal patterns and relationship between sensor-derived activity and sleep data, and clinically-relevant depressive states in this age range remains largely uncharted. Specifically, it is not clear to what extent findings from community samples align with those from clinical samples: addressing this point is important to build connections between actigraphic variables and pathophysiological processes, rather than individual differences across the spectrum of mood variation.

The present study is part of the larger Depression Early Warning (DEW) research project, aimed at the longitudinal mapping and modelling of the course of AMDD, including relapse prediction. The DEW project employs a combination of phenotypic measures, including passive measures such as wearable actigraphs (WA), active ecological momentary assessments (EMA), and in-person periodic clinical assessments.

Given its interim nature, this first analysis is not aimed at predicting MDD relapse by accelerometry: rather, given the paucity of this type of studies in youth, it explores the association between widely-adopted accelerometric indices, and measures of depression among youth who participate in a longitudinal study of MDD. Inasmuch as a recent study of adults [12] found both current and remitted MDD associated with variation in sleep and physical activity as indexed by the same technology employed in the DEW study, our aim was to assess whether same/similar findings could be substantiated in our longitudinal sample.

Therefore, here we: (a) gauged the interdependence among the WA variables of: Activity, Sleep Duration, and Sleep efficiency; (b) estimated by Latent Class Analysis (LCA) the number of classes that best capture the temporal unfolding of the WA variables in the longitudinal DEW sample, and: (c) assessed the ability of 2 widely-used clinically validated questionnaires for AMDD and its associated features - the Patient Health Questionnaire modified for adolescents (PHQ–A [22, 23]),, and the Ruminative Response Scale (RRS [24]), measured at baseline (i.e., at the DEW study intake), to predict WA class membership. Measurement over time can take a variable-centred approach (under the assumption of homogeneity), or a person-centred approach (under the assumption of heterogeneity of change). Here, by taking advantage of time-sensitive, dense sensor-derived data, we adopt a view of AMDD as a highly heterogeneous clinical phenotype that is best mapped with a person-centred approach.

We hypothesised that the WA variables of Activity, Sleep Duration, and Sleep efficiency would show sensible correlations, that individual patient-level data could be constrained into a finite number of meaningful latent classes, and that there would be associations between higher depressive and ruminative scores at baseline, and individual memberships into the different WA classes.

## Methods

### Setting

The DEW study is housed at the Centre for Addiction and Mental Health (CAMH) -an academic mental health provider- within the Child, Youth and Family Mood and Anxiety Team,

### DEW study recruitment and participants

Youth aged 12–21 with current or currently remitted clinically diagnosed DSM-5 MDD [25] were recruited for the DEW longitudinal digital phenotyping study. Participants are referred to the DEW study if among their presenting problems is a clinical diagnosis of ‘depression’ (both current and/or past). To be included in the study, participants had to meet the criteria for a current/past diagnosis of DSM-5 MDD, as assessed by a trained Research Coordinator with the DIAS-C structured psychiatric interview [26] (see below). Youth are excluded from participation into the DEW study if they have current or past substance use disorder whose severity is rated more than moderate by DSM-5 criteria, active psychotic symptoms, bipolar disorder, epilepsy, autism spectrum disorder, multiple sclerosis, paraplegia or spinal cord injury, juvenile rheumatoid arthritis or other major autoimmune disease, chronic renal failure, inherited metabolic disorders, or active cancer. Following clinicians’ referral, youth were recruited from: (a) the Mood and Anxiety clinic within CAMH’s Child, Youth and Emerging Adult Division, (b) online classified advertising including our hospital’s research recruitment page, and (c) referrals by professional health care providers in the greater Toronto area.

Participants were assessed for capacity to consent. Capacity of each potential participant to provide consent was assessed by a study team member trained by the PIs. Following the consent discussion, questions were asked to the participants to ensure they understood all study procedures, risks and benefits, as well as their rights as a volunteer in this research study. If the potential participant did not demonstrate such capacity during the informed consent process, the assumption of capacity to consent was not validated and the informed consent discussion did not continue. In this case, the potential participant was not considered eligible for the study.

In addition, non-CAMH recruited participants were asked for consent for DEW research personnel to contact their health professional to confirm their diagnosis of MDD, or having experienced an episode of MDD. CAMH participants’ diagnoses were confirmed through inspection of their medical charts.

### Data collection

Participants into the DEW study are administered clinical diagnostics at baseline, including: the DIAS-C (a semi-structured interview originally designed for familial-genetic studies that covers an extensive array of diagnoses) interview [26] to confirm their clinical MDD diagnosis. At baseline and at every following quarterly follow-up (named ‘arm’ in the present paper, taking place every 3 months), measures are collected via the following rating scales/questionnaires: the Patient Health Questionnaire adapted for adolescents (PHQ-9-A [22]),, and the Ruminative Response Scale (RRS [27, 28]). The PHQ-9-A consist of 9 items that correspond to the 9 DSM-IV criteria for MDD; it has comparable sensitivity and specificity to longer depression measures [22], and it was derived from the PHQ-9 [23], which is a popular clinical instrument to assess depressive symptoms both in psychiatric and in primary practice. The RRS was originally derived from the Response Style Questionnaire (RSQ [27]),, which included a self-report measure of rumination. A shorter, 8 item version of the RRS [24] showed good ability to measure within-person variation in rumination, so that a shorter version of the RRS was adopted in the DEW study too.

Following the administration of the aforementioned psychometric measures, 4 weeks of passive WA measurements are collected according to the DEW protocol. After the 4-week WA data collection, participants return the wearable device and complete the PHQ-9-A online using a REDCap survey [29]. The whole cycle of 4 weeks of passive data collection and PHQ administration is then repeated at every DEW quarterly follow-up.

The present report is based on the first 72 participants in the DEW study, recruited over the first 31 months of the study, and on the PHQ-9-A and the RRS scores filled at the recruitment of the DEW study (i.e., at baseline). Given the 2-year longitudinal nature of the DEW study, the maximum number of quarterly follow-ups is 8 (with baseline considered as point 1).

### Devices and technology

For each of the quarterly WA collections of the DEW study, the GENEActiv triaxial accelorometer original (Activinsights, Cambridge, United Kingdom) device is worn on the non-dominant wrist for 4 weeks (30 days) https://activinsights.com/technology/geneactiv/. The GENEActiv device has been validated against reference methods and proved reliable and valid by several studies [30–33]. Data were collected in person at CAMH, and Research staff were available to assist participants in clarifying questionnaires while completing study measures.

Geneactiv devices were set up in person with the participant. Geneactiv devices are lightweight and waterproof so that participants are able to wear them continuously throughout the day. Participants were given an instruction page about the device and were also encouraged to contact study staff if they had any problems with the devices, or if they were not able to wear them for the expected time frame. If participants contacted study staff, any issues or disruptions were noted.

Data related to sleep and activity were collected through self-report measures answered via mobile phones (EMA data) while participants were wearing the Geneactiv devices.

Data collected through the Geneactiv devices were verified by systematically checking: (a) the length of time participants wore the devices for (i.e., the consistency between the duration of data recording and the amount of time the device was worn); (b) near-body temperatures (these had to be *≥* 27 degrees Celsius as recorded by the Geneactive device, to ensure that the data received were a result of the participant’s actually wearing the device). In order to support the validity of Geneactiv data, nonconformities of sleep duration collected by the Geneactiv were also checked against self-reported EMA data, whereby participants were asked how many hours/day they had slept in any given day. Inconsistent matching between these sources led to data removal.

Whenever a discrepancy attributable to technical (e.g., battery insufficiently charged/dysfunctional) reasons was found, we adopted the Geneactiv Active Insights trouble shooting guide https://activinsights.com/technology/geneactiv/ to solve the issue.

The GGIR Package was used for processing WA data from GENEActiv devices and to extract actigraphy variables [33] listed in Table 1. Activity (in milligravity) was derived by the minute-level accelerometry count [12].

Further details about these parameters can be found in Appendix A.1.

Institutional Research Board (IRB) approval was granted by the CAMH Research Ethics Board.

**Table 1:** Variables used in the analyses

Variable	Source	Definition
Activity (in milligravity)	WA	Average acceleration
Sleep Duration (in minutes)	WA	Accumulated nocturnal sustained inactivity bouts within sleep period time
Sleep Efficiency (%)	WA	Ratio of the total sleep duration and sleep period time (difference between onset and waking time)
PHQ-A Score	REDCap	Score at baseline
RRS Score	REDCap	Score at baseline

*WA* wearable actigraph, *REDCap* secure web application for building and managing online surveys and databases

### Data analyses

Analyses were carried out with the R software version 1.4.1717 [34].

#### Aim 1: assessing correlation among WA features

We first assessed the correlations between WA variables (Activity, Sleep Duration, Sleep efficiency) by building a correlation matrix among the 3 WA variables’ grand means for each participant, with the *Hmisc* package in R [https://cran.r-roject.org/web/packages/Hmisc/index.html]. While this oversimplifies the picture by averaging within-individual variability in time, it provides a first, at-a-glance description of the correlations among-variables.

#### Aim 2: identifying trajectories using latent class analysis

In the light of the heterogeneity of the phenotype of MDD, to define classes of individuals with discrete psychophysiological patterns, we adopted LCA. This type of mixture modelling is often favoured in person-centred approaches like ours, as it is particularly apt at identifying latent subpopulations on the basis of their responses to observed variables. Specifically, LCA is suitable for identifying different classes within a heterogeneous population such as AMDD, and with complex data such as actigraphy data, in which patterns may not otherwise be easily identified.

We ran LCA models for each of the 3 WA variables using the *LCMM* Package in R ( https://cecileproust-lima.github.io/lcmm/ [34] across all available quarterly assessments. Models were run for solutions between 1 and 6 Latent Classes, with the selection of the best-fitting model based on a comprehensive evaluation of the following parameters: the Bayesian information criterion (BIC), Akaike Information Criterion (AIC) and Lo-Mendell-Rubin ad-hoc adjusted likelihood ratio test (calculated in R by the *calc_lrt* function), plus a criterion of parsimony, whereby no solutions were deemed acceptable if any cell (class) contained < 5% of subjects. The selected Latent class models were then visualized, with R code adapted from https://mcfromnz.wordpress.com/2011/10/02/latent-class-mixture-modeling-with-graphics/. The model in R can be described as follows: latent class model <- lcmm (dependent.variable ~ day + arm, mixture = ~ day, subject = participant.ID, ng = 2/3/4/5/6). Further details of these procedures can be found in Appendix B.

To estimate the degree of interdependence among the classes of the 3 WA variables, Cramer’s V coefficients were calculated using SPSS 27 [35].

#### Aim 3: predicting latent class membership through clinical questionnaires

We assessed the ability of PHQ-A’s scores at intake to predict membership into the WA classes by multinomial regression, where: (a) the PHQ-A scores at intake dichotomised into ‘clinical/non-clinical’ (PHQ-A > 14), and: (b) sex, were the predictors, and the most probable membership in every readout’s trajectory was the dependent variable, with age as a covariate.

Since rumination is a frequent associated feature of AMDD, we similarly assessed the ability of RRS scores at baseline to predict membership in WA class as the dependent variable by regression.

To corroborate our findings relative to Aims 2) and 3) (see below) via an approach that is alternative to LCA, we analysed the dataset with Linear mixed effects model [36], using random intercepts and the respective variables as fixed effects predictors. Differently from the LCA, this approach allows for estimating the effects of predictors on variation of accelerometry data per participant in time, without constraining individuals within any specified class.

For every accelerometry variable, we first ran an unconditional model, which only included the ‘arm’ and the ‘night’ variables as the predictors/covariates. Two versions of this unconditional model were run: one with the ‘arm’ variable treated as a linear time predictor (i.e., a version of the model that simply tested whether or not a significant temporal trend was discernible in the data), and another version that treated ‘arm’ as a semi-continuous predictor (i.e., a version of the model that tested whether one or more arms significantly differed from arm 1). Because of these features, one may consider these two versions of the unconditional model as nested one into the other. These two versions were compared one to the other, so that a successive conditional model with: age, sex, PHQ-9 (treated as a dichotomous variable: ‘clinical/non-clinical’ PHQ-A > 14), and RSS, could be run on the version of the model that provided the best fit. The ‘night’ variable was treated as a 30-level categorical covariate, but was not included in the summary.

Based on previous findings in community children [21] and in adults with MDD [12], we hypothesised associations between PHQ scores in the clinical range and: (a) reduced physical activity, and (b) shorter sleep duration.

## Results

### Sample

Over 31 months, we enrolled 72 individuals who provided WA data (72.5%% female, mean age in years: 17.44 *±* 2.2). Recruitment was carried out through clinician referral (*n* = 38), external advertisements (*n* = 25), referral from other research studies (*n* = 6), and our hospital’s research website (*n* = 3). Preliminary analyses showed that the mean age and sex of clients with missing data did not differ significantly between hospital recruitment and external recruitment sources. According to the diagnostic interviews, 44 (61%) participants had a current MDD episode, while the remaining participants were in remission. The number of quarterly collections varied between 1 and 7 (as no one had yet completed the entire quarters of this 2 year longitudinal study), with the median number of collections in WA being 2.51.

### Aim 1: assessing covariation among WA features

Table 2 shows the correlations among the 3 WA-generated variables in the study, obtained by averaging the means of each participant for all arms of the study. While these values are insensitive to change in time, they provide a broad picture of the interrelationships among variables. In examining the correlations, it is important to remember that Sleep Efficiency is the % ratio between the total sleep duration and sleep period time: as such, its correlation with Sleep Duration is non-independent. Sleep Efficiency showed moderate, positive correlations with Acceleration, indicating positive covariation between actigraph-measured sleep efficiency and the amount of physical activity. Sleep Duration appeared only weakly and negatively correlated with Acceleration.

**Table 2:** Grand mean correlations among the 3 actigraphic (WA) variables

Correlation	Acceleration	Sleep duration	Sleep efficiency
Acceleration	-		
Sleep duration	−0.15	-	
Sleep efficiency	0.31*	0.62**	-

Each observation of the data set was the averaged means of each participant for all arms (between 1 and 8) within the study

The Cramer V coefficient between acceleration and sleep efficiency was 0.27 (*p* = 0.04), and 0.21 (*p* = 0.17) between acceleration and sleep duration

*p < 0.05 **p < 0.001

### Aim 2: identifying trajectories using latent class analysis

**Fig. 1:**
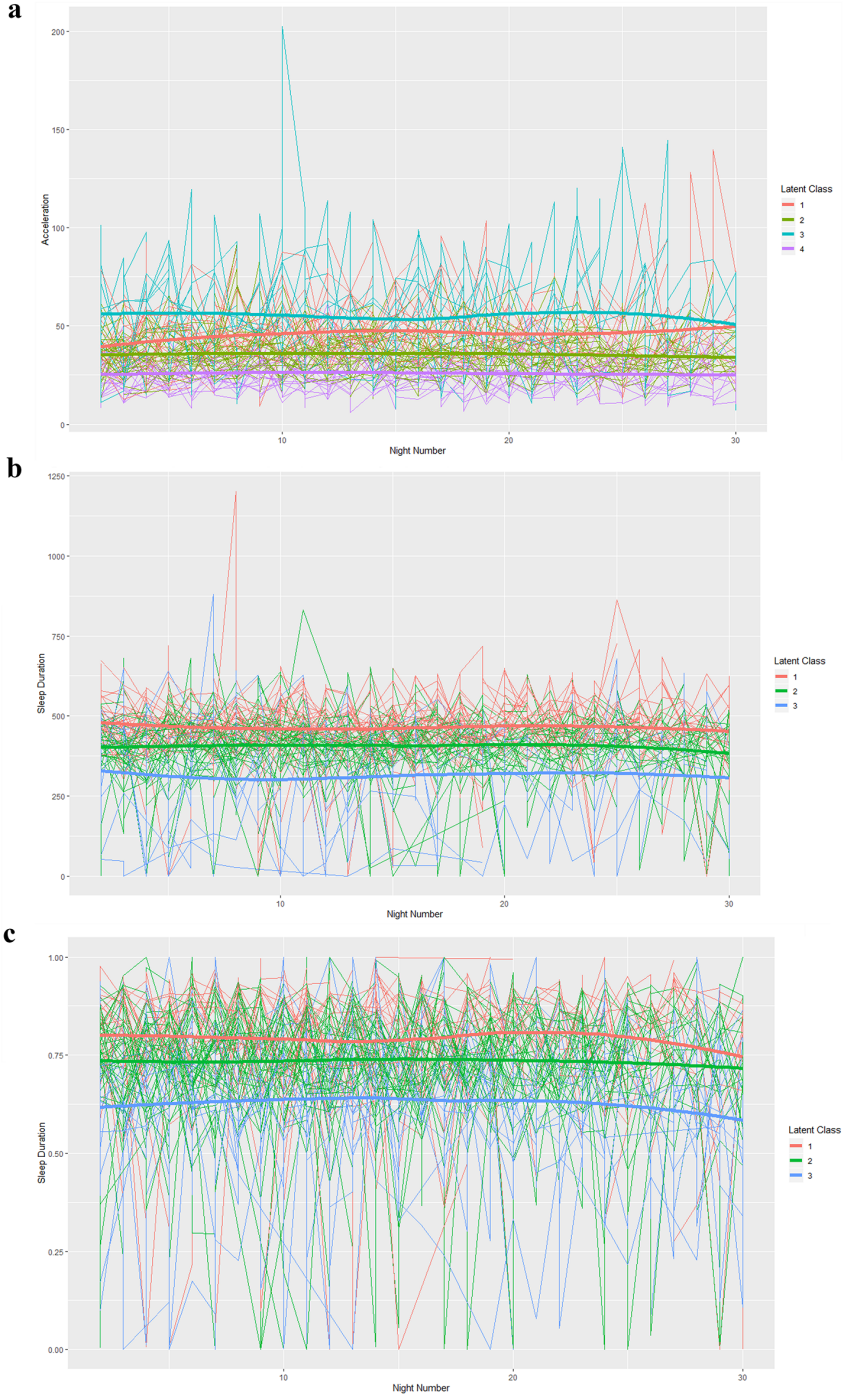
Visualisation of best-fitting latent class analyses of acceleration (Activity), sleep duration, sleep efficiency. **a** Average Activity (Acceleration in mg) over Days (1–30) within each arm for model with 4 latent classes. **b** Sleep Duration (min) over Days (1–30) within each arm for model with 3 latent classes. **c** Sleep Efficiency (%) over Days (1–30) within each arm for model with 3 latent classes

Table 3; Fig. 1 show the results of latent class analyses for WA-generated variables: between 3 and 4 classes were required to achieve an adequate fit to the models.

**Table 3:** Latent class analysis model summary for WA variables (N subjects = 72, N observations = 3716, with best fitting solution in bold) % subjects in class

Variable	*N* of Classes	Log Likelihood	BIC	AIC	1	2	3	4	5	6
Acceleration	1	−15757.1	31544.1	31528.2	100.0					
	2	−15304.7	30652.1	30629.3	37.5	62.5				
	3	−15205.6	30466.7	30437.1	18.1	52.8	29.2			
	4*	**−15154.5**	**30377.4**	**30341.0**	**23.6**	**41.7**	**11.1**	**23.6**		
	5”	−15166.1	30413.4	30370.1	9.7	22.2	40.3	22.2	5.6	
	6	−15138.1	30370.3	30320.2	23.6	37.5	11.1	11.1	1.4	15.3
Sleep duration	1	−22589.1	45208.1	45192.2	100.0					
	2	−22417.5	44877.7	44854.9	86.1	13.9				
	**3**	**−22336.4**	**44728.3**	**44698.7**	**41.7**	**47.2**	**11.1**			
	4	−22251.9	44572.3	44535.9	34.7	51.4	11.1	2.8		
	5*	−22234.2	44549.7	44506.4	51.4	34.7	6.9	5.6	1.4	
	6	−22226.5	44547.0	44497.0	34.7	51.4	6.9	4.2	1.4	1.4
Sleep efficiency	1	2133.4	−4215.6	−4242.9	100.0					
	2	2340.9	−4617.7	−4651.9	75.0	25.0				
	3*	**2400.2**	**−4723.4**	**−4764.4**	**27.8**	**54.2**	**18.1**			
	4	2405.6	−4721.5	−4769.3	12.5	23.6	18.1	45.8		
	5	2414.6	−4726.5	−4781.2	27.8	52.8	5.6	8.3	5.6	
	6	2414.7	−4713.9	−4775.3	27.8	45.8	18.1	2.8	5.6	0.0

Selected latent class models based on BIC, AIC, Lo-Mendel test and class distributions

*Lo-Mendell-Rubin ad-hoc adjusted likelihood ratio test results not significant (at alpha = 0.05) between class # and the next (e.g. *p* = 1.00 between 4 and 5 classes)

Model failed to converge at global maximum, hence Log Likehood is smaller than previous class

Overall all these classes showed steady, relatively flat trajectory shapes (Fig. 1) that indicate time stability, within the limited time of observation of this analysis. Figure 1 (a to c) also shows remarkable variance around the aforementioned trajectories.

### Aim 3: predicting latent class membership through clinical questionnaires

The multinomial regression model for Acceleration (4 classes) yielded a significant overall model result (Chi-square = 22.5, DF = 12, *p* = 0.03), with a significant effect for PHQ-A score (Likelihood ratio chi square = 9.8, DF = 3, *p* = 0.02) yielding Wald = 6. *p* = 0.014, Exp. B = 20.1, with a prediction of Trajectory 1 vs. trajectory 4 in the expected direction. This means that less Acceleration was predicted by higher (i.e., > 14) PHQ-A at baseline. Neither sex nor age significantly affected the association.

The multinomial regression model for Sleep Duration (3 classes) yielded a significant overall model result (Chi-square = 15.6, DF = 6, *p* = 0.016), a Cox-Snell pseudo R square = 0.23 and a significant effect for PHQ–A score (Likelihood ratio chi square = 6.6, DF = 2, *p* = 0.04), with a prediction of Trajectory 1 vs. trajectory 3 in the expected direction. This means that shorter sleep duration was predicted by higher (i.e., > 14) PHQ-A at baseline. Neither sex nor age significantly affected the association.

The multinomial regression model for Sleep Efficiency (3 classes) yielded a non-significant overall model result (Chi-square = 12.1, DF = 6, p = NS). Similarly, regression models to predict class membership by RRS score did not yield significant results.

Linear mixed effects model.

- Acceleration

For Acceleration, both version of the unconditional model yielded significant results, but comparison between the two versions (with DF respectively = 3615 and 3612) showed better fit (likelihood ratio test chi-square = 24.00, *p* < 0.001) for the version with ‘arm’ as a semi-continuous predictor. When a conditional model with: age, sex, PHQ-9, and RSS, was run on the latter model, and showed a significant effect only age (*p* = 0.007) and suggestive evidence for PHQ (*p* = 0.057). See detailed model summary in Table 4.

(b) Sleep duration

For Sleep Duration, the results of linear mixed models resembled those obtained with Acceleration, in that a conditional model with ‘arm’ as a semi-continuous predictor and age, sex, PHQ, and RSS, showed an effect for PHQ (*p* = 0.03) only. However, a sensitivity analysis with all high influential cases deleted reduced the significance of PHQ (*p* = 0.06).

(c) Sleep efficiency

Both unconditional models for Sleep Efficiency yielded non-significant results, so that their statistical comparison was meaningless. When we ran a model with ‘arm’ as a semi-continuous predictor and age, sex, PHQ-9, and RSS, no significant effect emerged.

**Table 4:** Detailed summary of linear mixed-effects model output

Outcome	Predictor^1^	$\:\beta\:$ estimate	Standard error	Degrees of freedom	t-value	95% CI	*p*-value	Effect size
Acceleration^4^	Intercept	64.202	8.821	3119	7.278	(47.006, 81.398)	< 0.001	
	Male vs. female	2.894	2.661	56	1.088	(−2.406, 8.194)	0.281	0.176
	Age	−1.322	0.469	56	−2.816	(−2.257, −0.387)	0.007	−0.081
	PHQ-9	−4.827	2.479	56	−1.947	(−9.765, 0.111)	0.057	−0.294
	RRS	−0.109	0.316	56	−0.343	(−0.739, 0.522)	0.733	−0.007
	Arm 2 vs. arm 1^2^	−2.516	0.688	3119	−3.66	(−3.857, −1.176)	< 0.001	
	Arm 3 vs. arm 1	−0.375	0.775	3119	−0.484	(−1.886, 1.136)	0.629	
	Arm 4 vs. arm 1	−2.555	0.931	3119	−2.745	(−4.369, −0.74)	0.006	
	Arm 5 vs. arm 1	−7.112	1.608	3119	−4.423	(−10.247, −3.977)	< 0.001	
Sleep duration^5^	Intercept	477.712	64.552	3122	7.4	(351.815, 603.608)	< 0.001	
	Male vs. female	−2.244	3.435	56	−0.653	(−9.088, 4.6)	0.516	−0.02
	Age	20.474	19.486	56	1.051	(−18.355, 59.302)	0.298	0.182
	PHQ-9	40.953	18.125	56	2.259	(4.837, 77.07)	0.028	0.364
	RRS	−2.586	2.314	56	−1.117	(−7.198, 2.025)	0.269	−0.023
	Arm^3^	1.756	1.709	3122	1.028	(−1.577, 5.089)	0.304	

^1^The night variable (30 levels) was included in the model as a categorical predictor but their estimates are not included here

^2^Arm was treated as a categorical predictor since it improves the fit significantly (p-value < 0.001) in contrast to being treated as a semi-continuous predictor

^3^Arm was treated as a semi-continuous predictor since being treated as a categorical predictor did not improve the fit significantly (p-value = 0.292)

^4^Model diagnostic analysis did not show deviation from the normality assumption (p-value = 0.353, Kolmogorov-Smirnov Test). About 5.4% of the observations were categorized as high influential cases. However, a sensitivity analysis with these cases deleted did not show substantial differences

^5^Model diagnostic analysis showed deviation from the normality assumption (p-value = 0.002, Kolmogorov-Smirnov Test). Given the large sample size, this has little impact on the validity of the tests for significance. About 5.3% of the observations were categorized as high influential cases. A sensitivity analysis with these cases deleted changed the significance of PHQ-9 with a p-value of 0.061

## Discussion

This study shows that: a) the WA readouts show reciprocal, sensible correlations; b); a dense amount of longitudinally-assessed, wearable-collected data relative to: Acceleration, Sleep Duration, Sleep Efficiency is reducible into a finite number of classes c) membership into classes of higher vs. lower physical activity (as indexed by Acceleration), and shorter vs. longer Sleep Duration are predicted by the PHQ-A, a popular measure of depression in youth.

The correlation matrix of averaged: Acceleration, Sleep Duration, and Sleep Efficiency showed moderate-to-substantial reciprocal correlations in the expected directions, in that greater Acceleration was associated with greater Sleep Efficiency. In addition, we found the same modest, negative, association between Acceleration and Sleep Duration found in a cohort of US adults [6] assessed with the GENEActiv device. Overall, the correlation matrix indicated that amongst youth at varying clinical stages of AMDD, more physical activity is associated with better sleep quality, which constitutes a validation through objective, longitudinally-gathered WA measures, of a similar conclusion yielded by a systematic review of 141 studies of adolescents across 57 different countries [8].

The insights from the time-insensitive approximations yielded by the correlation matrix were then better specified and expanded in the context of the latent class analyses, which allowed for a more granular examination of the temporal unfolding of the WA variables, and of their reciprocal relationships. The WA longitudinal data organised into 3 accelerometer measures could be fit into a finite number of clearly separated classes (specifically: 3 classes for Sleep Duration and Sleep Efficiency, and 4 classes for Acceleration). This finding is qualitatively similar to the results obtained by wearable-assisted longitudinal assessments of general population English adolescents [21] that showed 3 distinct and stable subgroups: light activity, moderate-to-vigorous activity, sedentary.

Our LCA modelling (Fig. 1, a through c) also yielded a clear degree of temporal stability across all the classes of Acceleration, Sleep Duration, and Sleep Efficiency, again in keeping with the 4 year longitudinal study with accelerometers of the ALSPAC adolescents, between age 12 and age 16 [21]. This altogether suggests that the psychophysiological variables of Acceleration, Sleep Duration, and Sleep Efficiency as captured by actigraphy reflect relatively stable sources of individual differences, and may be considered biopsychological traits against which the depressive symptomatology can be juxtaposed, and mapped. In addition to this joint variation, in keeping with studies of WA in adults [12], our data also show a degree of within-classes individual variation. While we did not directly address individual variation in our latent classes, multimodal data-reduction technique showed that that WA’s joint components’ variation outweighs individual variation.

Our third aim addressed the relationships between membership in WA classes and: PHQ-A scores, and RRS score at baseline. The significant association between a clinical PHQ-A score (PHQ-A > 14) and the trajectory of lowest physical activity (Acceleration) is in broad keeping with the results of a mendelian randomisation study of adults with MDD that showed a protective relationship between accelerometer-based activity and MDD [17].

The mixed model analyses yielded results that were consistent with our LCA findings, in that PHQ showed some effect for both Acceleration and Sleep Duration. This implicates that individuals who entered the study with clinical symptoms of depression tend to show relatively stable patterns of lower physical activity and shorter sleep duration across multiple quarterly checkpoints in up to 2 year timeline. This should be best considered as a suggestive evidence, and future validation of this early finding is warranted.

In keeping with the results of the START longitudinal study of depression in adolescents [16], a score above clinical cut-off on the PHQ-A predicted shorter Sleep Duration. These results indicate a relatively robust, replicable relationship between objective measures of physical activity, sleep and depressive symptoms among adolescents diagnosed with MDD, and resonate with the notion that physical activity is beneficial for/protective against AMDD [18]. They also support targeting behavioural activation as a treatment for AMDD [18]. The lack of significant covariation with ruminative scores may in turn indicate a relative specificity of the above-mentioned relationships, in that the PHQ-A scores that directly tap the construct of AMDD, but not the RRS scale that assesses the AMDD-associated feature of rumination, predict specific classes of low physical activity and shorter sleep duration. It may also suggest that rumination has a limited role in the physical experiences (sleep disturbance, physical activity) of AMDD, supporting the use of activation approaches more than addressing rumination or other cognitive facets among youth who experience physical, more than cognitive symptoms of AMDD.

A certain amount of individual variability also emerges from the temporal unfolding of Acceleration, Sleep Duration, and Sleep Efficiency (Fig. 1, a through c). As the DEW study sample grows in time, and more data become available, this temporal variation will become available to statistically more powerful and reliable analyses. These future analyses can encompass potentially relevant variables such as: seasonality, day-of-the-week (i.e. week-ends vs. school days), menstrual cycles, etcetera.

In conclusion, our data provide evidence in favour of significant and clinically meaningful covariations among classes of Acceleration, Sleep Duration and self-assessed symptoms of depression; the underlying causal mechanisms of this covariation are however still being investigated. Our findings align to a considerable extent with those of similar investigations carried out among adolescents of the ALSPAC cohort [21] and adults with/at risk for MDD [12], and support the view that at least some of the accelerometric variables are biomarkers of MDD. The evidence in favour of considerable, joint variation among accelerometric variables suggests that this shared source of variation can be the target of future etiological and clinical studies of MDD.

### Limitations

This is an interim study based on a relatively small sample: as such, it likely yields a limited power and will need extension from further recruitment of participants with the DEW study cohort, as well as replication from other groups. The data collection was limited to quarterly collections of 30 days via wearables. This implies a lack of data points -hence potential loss of relevant information- between the different assessment periods. However, as with any studies that imply longitudinal assessments and the adoption of technology [37, 38], we needed to strike the balance between density of data collection, GeneActiv’s battery autonomy, and the potential impact of prolonged use of wearable on participants’ compliance. Recent studies that employed the same device in adults managed to extract meaningful data on accelerometry variation and MDD from 14 days of data collections [12]. The association between depressive scores and physical activity could be explained by confounders that were not taken into account here. However, analyses carried out in large adult samples that took into account several confounders (such as: race, BMI, medication) still found a sizable, reliable degree of accelerometry-based variability and MDD [12].

Moreover, given the complexity and density of the data, it appears that new approaches such as machine learning [39] can improve our ability to analyse wearable-collected data, obtain more eloquent patterns of association, and potentially derive powerful models of relapse.

## Conclusions

Wearable-generated variables on physical activity and sleep can be reduced to a finite number of classes of individuals; interfacing membership in these classes with clinical measures of adolescent depression yield statistically significant and clinically meaningful relationships. The conjoint use of objective, wearable-generated measures and of clinical measures of depression may deepen our understanding of AMDD. The identification of distinct classes of people with specific patterns of physical activity and sleep can inform more focused interventions on AMDD. For instance, membership in a class of low physical activity would call for more emphasis on activation therapy, while membership in a class of shorter sleep duration would suggest more in-depth therapeutic work in sleep hygiene.

## Supplementary Information

Additional file1 (DOCX 30 kb)

## Data Availability

Data is provided within the manuscript or supplementary information files.
