# Supplementary material for: Latent class analysis of actigraphy within the depression early warning (DEW) longitudinal clinical youth cohort
Source: Child Adolesc Psychiatry Ment Health. 2024 Nov 19;18:149. doi: 10.1186/s13034-024-00843-8 (PMC11577627; doi:10.1186/s13034-024-00843-8)
Supplement: Supplementary file 1 — Additional file1 (DOCX 30 kb) [file 13034_2024_843_MOESM1_ESM.docx]

**Appendix**

**Appendix A. Details on parameters for extracting WA variables**

g.shell.GGIR(#-------------------------------

# General parameters

#-------------------------------

mode = mode,

datadir = datadir,

outputdir = outputdir,

studyname = studyname,

f0 = f0,

f1 = f1,

overwrite = TRUE,

do.imp = TRUE,

idloc = 1,

print.filename = FALSE,

storefolderstructure = FALSE,

#-------------------------------

# Part 1 parameters:

#-------------------------------

windowsizes = c(5,900,3600),

do.cal = TRUE,

do.enmo = TRUE,

do.anglez = TRUE,

chunksize = 1,

printsummary = TRUE,

desiredtz= "America/Toronto",

#-------------------------------

# Part 2 parameters:

#-------------------------------

strategy = 1,

ndayswindow = 7,

hrs.del.start = 1,

hrs.del.end = 1,

maxdur = 9,

includedaycrit = 16,

L5M5window = c(0,24),

M5L5res = 10,

winhr = c(5,10),

qlevels = c(c(1380/1440),c(1410/1440)),

qwindow = c(0,24),

ilevels = c(seq(0,400,by = 50),8000),

mvpathreshold = c(100,120),

#-------------------------------

# Part 3 parameters:

#-------------------------------

timethreshold = c(5,10),

anglethreshold = 5,

ignorenonwear = TRUE,

#-------------------------------

# Part 4 parameters:

#-------------------------------

excludefirstlast = FALSE,

includenightcrit = 16,

def.noc.sleep = c(),

loglocation = c(),

outliers.only = FALSE,

criterror = 4,

relyonsleeplog = FALSE,

sleeplogidnum = TRUE,

colid = 1,

coln1 = 2,

do.visual = TRUE,

nnights = 9,

#-------------------------------

# Part 5 parameters:

#-------------------------------

# Key functions: Merging physical activity with sleep analyses

threshold.lig = c(30,40,50),

threshold.mod = c(100,120),

threshold.vig = c(400,500),

excludefirstlast = FALSE,

boutcriter = 0.8,

boutcriter.in = 0.9,

boutcriter.lig = 0.8,

boutcriter.mvpa = 0.8,

boutdur.in = c(10,20,30),

boutdur.lig = c(1,5,10),

boutdur.mvpa = c(1,5,10),

timewindow = c("WW"),

#-----------------------------------

# Report generation

#-------------------------------

do.report = c(2,4,5),

visualreport = TRUE, dofirstpage = TRUE,

viewingwindow = 1)

## End(Not run)

**Appendix B.**

**Selected latent class models based on BIC, AIC, Lo-Mendel Test and Class distributions for WA variables**

- Fixed effects: a two-sided linear formula object for specifying the fixed-effects in the linear mixed model at the latent process level. The response outcome is on the left of ~ and the covariates are separated by + on the right of the ~.
  Arm = factor variable
- Mixture effects: a one-sided formula object for the class-specific fixed effects in the latent process mixed model (to specify only for a number of latent classes greater than 1). Among the list of covariates included in fixed, the covariates with class-specific regression parameters are entered in mixture separated by +.
  By default, an intercept is included. If no intercept, -1 should be the first term included.
- Subject: name of the covariate representing the grouping structure.
- ng: number of latent classes considered

Appendix C

Parameters distribution across the 3 accelerometer variables

| Average Activity |  | Class 1 (n = 7) | Class 2 (n = 19) | Class 3 (n = 27) | Class 4(n = 19) | $\chi^{2}$   \|  \| \| --- \| | p-value |
| --- | --- | --- | --- | --- | --- | --- | --- | --- |
| age |  | 15.57 ± 3.15 | 16.63 ± 2.85 | 17.59 ± 2.15 | 18.63 ± 2.34 | 8.59 | 0.035 |
| sex | F | 3(42.9%) | 13(68.4%) | 23(85.2%) | 14(73.7%) | 5.53 | 0.131 |
|  | M | 4(57.1%) | 6(31.6%) | 4(14.8%) | 5(26.3%) |  |  |
| PHQ_raw |  | 14 ± 5.8 | 11.86 ± 6.7 | 12.86 ± 5.44 | 16.11 ± 7.67 | 4.39 | 0.222 |
| PHQ_coded | 0 | 4(57.1%) | 7(50%) | 14(63.6%) | 4(22.2%) | 7.2 | 0.063 |
|  | 1 | 3(42.9%) | 7(50%) | 8(36.4%) | 14(77.8%) |  |  |
| RRS SUM |  | 15.57 ± 6.19 | 16.89 ± 3.81 | 17.07 ± 3.94 | 18.11 ± 3.53 | 1.64 | 0.651 |

| Sleep Duration |  | Class 1 (n = 31) | Class 2 (n = 34) | Class 3 (n = 7) | $\chi^{2}$   \|  \| \| --- \| | p-value |
| --- | --- | --- | --- | --- | --- | --- | --- |
| age |  | 17.39 ± 2.53 | 17.24 ± 2.83 | 18.43 ± 2.07 | 1.15 | 0.564 |
| sex | F | 25(80.6%) | 22(64.7%) | 6(85.7%) | 2.71 | 0.331 |
|  | M | 6(19.4%) | 12(35.3%) | 1(14.3%) |  |  |
| PHQ_raw |  | 14.92 ± 7.1 | 13.79 ± 6.14 | 8.17 ± 2.64 | 6.76 | 0.034 |
| PHQ_coded | 0 | 10(38.5%) | 13(44.8%) | 6(100%) | 7.57 | 0.022 |
|  | 1 | 16(61.5%) | 16(55.2%) | 0(0%) |  |  |
| RRS SUM |  | 16.94 ± 3.88 | 17.53 ± 4.28 | 16.29 ± 3.82 | 1.17 | 0.558 |

| Sleep Efficiency |  | Class 1 (n = 39) | Class 2 (n = 26) | Class 3 (n = 7) | $\chi^{2}$   \|  \| \| --- \| | p-value |
| --- | --- | --- | --- | --- | --- | --- | --- |
| age |  | 16.72 ± 2.61 | 18.19 ± 2.42 | 18.43 ± 2.7 | 5.97 | 0.056 |
| sex | F | 29(74.4%) | 19(73.1%) | 5(71.4%) | 0.03 | 1 |
|  | M | 10(25.6%) | 7(26.9%) | 2(28.6%) |  |  |
| PHQ_raw |  | 12.54 ± 7.12 | 15.35 ± 6.04 | 12.43 ± 5.44 | 1.94 | 0.379 |
| PHQ_coded | 0 | 14(50%) | 11(42.3%) | 4(57.1%) | 0.61 | 0.767 |
|  | 1 | 14(50%) | 15(57.7%) | 3(42.9%) |  |  |
| RRS SUM |  | 17.08 ± 4.11 | 17.69 ± 3.76 | 15.57 ± 4.72 | 1.06 | 0.59 |
